# Modeling for Predicting the Potential Geographical Distribution of Three *Ephedra* Herbs in China

**DOI:** 10.3390/plants12040787

**Published:** 2023-02-09

**Authors:** Longfei Guo, Yu Gao, Ping He, Yuan He, Fanyun Meng

**Affiliations:** 1 Beijing Key Laboratory of Traditional Chinese Medicine Protection and Utilization, Faculty of Geographical Science, Beijing Normal University, Beijing 100875, China; 2 State Key Laboratory of Earth Surface Processes, Faculty of Geographical Science, Beijing Normal University, Beijing 100875, China; 3 Engineering Research Center of Natural Medicine, Ministry of Education, Faculty of Geographical Science, Beijing Normal University, Beijing 188875, China

**Keywords:** ensemble model, *Ephedra*, suitable habitat, species protection

## Abstract

*Ephedra* species are beneficial for environmental protection in desert and grassland ecosystems. They have high ecological, medicinal, and economic value. To strengthen the protection of the sustainable development of *Ephedra*, we used occurrence records of *Ephedra sinica* Stapf., *Ephedra intermedia* Schrenk et C.A. Mey., and *Ephedra equisetina* Bge., combined with climate, soil, and topographic factors to simulate the suitable habitat of three *Ephedra* based on ensemble models on the Biomod2 platform. The results of the models were tested using AUC, TSS, and kappa coefficients. The results demonstrated that the ensemble model was able to accurately predict the potential distributions of *E. sinica*, *E. intermedia*, and *E. equisetina*. Eastern and central Inner Mongolia, middle and eastern Gansu, and northeastern Xinjiang were the optimum regions for the growth of *E. sinica*, *E. intermedia*, and *E. equisetina*, respectively. Additionally, several key environmental factors had a significant influence on the suitable habitats of the three *Ephedra*. The key factors affecting the distribution of *E. sinica*, *E. intermedia*, and *E. equisetina* were annual average precipitation, altitude, and vapor pressure, respectively. In conclusion, the results showed that the suitable ranges of the three *Ephedra* were mainly in Northwest China and that topography and climate were the primary influencing factors.

## 1. Introduction

*Ephedra* species are perennial herbs of the *Ephedra* genus in Ephedraceae. Members of this genus are commonly found in dry wastelands, riverbeds, and grasslands. They are important species associated with sandy grassland and frequently form a large area of a simple community [1]. These plants are important sand fixation plants in Northwest China and assist with water and soil conservation, desertification prevention, and environmental improvement because of their high drought tolerance and ability to withstand extreme temperature and saline alkali [2]. Fifteen *Ephedra* species and four varieties can be found in China, and *E. sinica*, *E. intermedia*, and *E. equisetina* are the main varieties used in medicine [3]. Three *Ephedra* herbs have been widely used as traditional Chinese medicine (TCM) since ancient times [4] and are used to primarily treat colds, coughs, and asthma [5]. The medicinal effect of *Ephedra* cannot be achieved without active substances. *Ephedra* herbs contain a variety of alkaloids, volatile substances, flavonoids, sugars, minerals, among which the content of alkaloids is the highest, and alkaloids are the main active components of *Ephedra* [6]. Modern pharmacological studies have revealed that *Ephedrine* has the functions of favoring sweating, alleviating colds, promoting lung health, relieving asthma, and detumescence. Many effects of *Ephedra* are associated with ephedrine-type alkaloids. The effects of various secondary metabolites differ. For example, ephedrine is an effective component for ephedra to play analgesic, regulate blood pressure, stimulate the central nervous system, and relax bronchial smooth muscle [7,8,9,10]. Pseudoephedrine is an effective component for ephedra to play diuretic and anti-inflammatory [11,12]. However, the alkaloid contents showed certain differences among the three species, so we need to predict the suitable habitat of the three ephedra species respectively. In addition, as an important forage grass, *Ephedra herbs* have edible and medicinal properties, can effectively prevent livestock diseases, and promote the development of local animal husbandry [13]. Clearly, *Ephedra* herbs have tremendous ecological, medicinal, and economic value. However, there has been a rapid increase in the demand for *Ephedra* herbs on the international market in recent years, and their price and economic benefits have also increased, resulting in an increase in destructive harvesting. The scope of *Ephedra* species distribution has been greatly reduced, resulting in significant grassland degradation as well as water and soil erosion. Grassland and desert ecosystems have been harmed, affecting the normal production and way of life of farmers and herders [14]. Research on the suitable habitat of *Ephedra* herbs contributes to the conservation of their resources and the restoration of their ecosystems [15,16]. As a result, it is necessary to investigate the potential distribution and explore the ecological suitability of *Ephedra* herbs. Because the alkaloid contents of these three *Ephedra* herbs differ, so do their medicinal value. It is necessary to predict the potential distributions of *E. sinica*, *E. intermedia*, and *E. equisetina*.

Species distribution models (SDMs) are frequently used in the intersection of geography, ecology, and species conservation. They are effective tools for investigating ecological issues related to species and the environment [17,18], and they are widely used in the fields of endangered species protection [19,20] and environmental impact assessment [21]. SDMs primarily use species distribution data and environmental data to estimate a species’ ecological niche and project it into the landscape to reflect the species’ preference for habitat in the form of probability. The results of SDMs can be interpreted as species occurrence probability, habitat suitability, or species richness [22,23,24]. Most studies currently use single SDMs, such as the maximum entropy model (MaxEnt), Bioclim model, and genetic algorithm for the rule-setprediction model (GARP) [25,26,27], to predict a species potential habitat. Semwal et al. [28] used the Bioclim model to predict the distribution of *makhana* (*Euryale ferox* Salisb.) in the Indian region. Based on the 19 bioclim data and occurrence records, they predicted that the areas highly suitable for *makhana* shifted from major parts in Jharkhand, Uttar Pradesh, and West Bengal to negligible areas bordering the Katihar district of Bihar. Zhong et al. [29] predicted the distribution of *Wedelia trilobata* (L.) Hitchc., an invasive plant in many regions, by using MaxEnt and GARP models. After comparing the results of the two models, they found that the MaxEnt model findings were more conservative and that GARP had the greater predictive capability. However, the robustness of a single-species model will continue to decline as input data variation increases [30]. This situation can be effectively avoided using ensemble models. Aguirre-Gutiérrez et al. [31] used six single SDMs and an ensemble model to predict the distribution area of Dutch hoverflies and evaluated each model in terms of the area under the curve (AUC), the geographical and spatial consistency of the prediction results and the consistency of the selection and contribution of environmental variables. They found that the ensemble model was obviously better at predicting species distribution than single models. Biomod2 is an ensemble modeling platform based on R language that sets ten common single models, including Artificial neural networks (ANN), Classification tree analysis (CTA), Flexible discriminant analysis (FDA), Generalized additive model (GAM), Generalized Boosting Model or Boosted Regression Trees (GBM/BRT), Generalized linear model (GLM), Multivariate adaptive regression splines (MARS), MaxEnt, Random forest (RF), Surface range envelope (SRE). By constructing ensemble models and integrating the different principles, assumptions, and algorithms of different models, the platform avoids the instability of single model simulation and realizes high-precision prediction [32,33]. As the most mature multimodel platform currently, the Biomod2 platform can better simulate the spatial distribution of species and infer its main influencing factors, and thus, this model has been recognized and used by many scholars [34,35]. Uusitalo et al. [36] predicted the distribution of mosquitoes in genera *Culex* L. and *Stegomyia* Theobald in the Taita Hills, southeastern Kenya, by using Biomod2, and the results showed that factors including population density, road distance, and slope had a greater impact on *Culex*, while population density, solar radiation, temperature, and vegetation had a greater impact on *Stegomyia*. Resquin et al. [37] used Biomod2 modeling to investigate the current and potential habitat distribution of two types of *eucalyptus* forests, *Eucalyptus grandis* Hill ex Maiden and *E. dunnii* Maiden in Uruguay, and discovered that soil surface depth was the most important factor affecting the distribution of these two species.

The Biomod2 platform was used in this study to construct ensemble models for *E. sinica*, *E. intermedia*, and *E. equisetina*. Based on the ensemble models, we predicted the distribution of the three species in China, clarified the impact mechanism of the potential habitat pattern, and provided a theoretical basis for an in-depth understanding of *Ephedra* resource sustainability. Our study had three objectives: 1. explore the main limiting factors of three *Ephedra* herbs distributions; 2. identify the important suitable habitat areas for *E. sinica*, *E. intermedia*, and *E. equisetina*; and 3. distinguish priority sites for *Ephedra* species conservation and reforestation.

## 2. Results

### 2.1. The SDM and Its Accuracy

The average AUC, TSS, and kappa values of single models and ensemble models for three *Ephedra* species were evaluated on the Biomod2 platform (Table 1). Among the 10 single models, RF, GBM, and GLM had high accuracy and excellent performance in predicting the potential distribution areas of *E. sinica*, *E. intermedia*, and *E. equisetina*, while SRE had the worst accuracy. The average AUC, TSS, and kappa values for *E. sinica*’s ensemble model were 0.97, 0.84, and 0.79, respectively; those for *E. intermedia* were 0.98, 0.87, and 0.81, respectively; and those for *E. equisetina* were 0.98, 0.91, and 0.87, respectively. Compared with single models, the ensemble models for three *Ephedra* species had significantly improved AUC, TSS, and kappa values, indicating that the ensemble models could more accurately predict potentially suitable habitats for *E. sinica*, *E. intermedia*, and *E. equisetina*.

### 2.2. Important Environmental Variables

**Figure 1:**
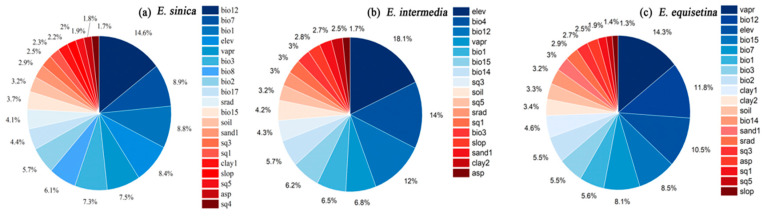
Percentage contributions of the environmental variables in the ensemble models for *E. sinica* (**a**), *E. intermedia* (**b**), and *E. equisetina* (**c**).

According to the contribution degree of environmental variables to *E. sinica*, *E. intermedia*, and *E. equisetina* in the ensemble models (Figure 1), annual precipitation (bio12, contributing 14.9%) had the greatest impact on the distribution of *E. sinica*, followed by the annual temperature range (bio7, 8.9%), annual mean temperature (bio1, 8.8%), and elevation (elev, 8.4%) (Figure 1a). Furthermore, elevation (elev, 18.1%) had the greatest impact on the distribution of *E. intermedia*, followed by temperature seasonality (bio4, 14%) and annual average precipitation (bio12, 12%) (Figure 1b); the environmental variable with the greatest driver for the distribution of *E. equisetina* was vapor pressure (vapr, contributing 14.3%), followed by mean annual precipitation (bio12, 11.8%), elevation (elev, 10.5%), coefficient of variation of precipitation (bio15, 8.5%) and temperature annual range (bio7, 8.1%) (Figure 1c). In general, climate variables and topographic variables were the main environmental variables affecting the distribution of *Ephedra* herbs, while soil variables had relatively little influence.

### 2.3. Potential Suitable Area Distribution for Three Ephedra Herbs Species

**Figure 2:**
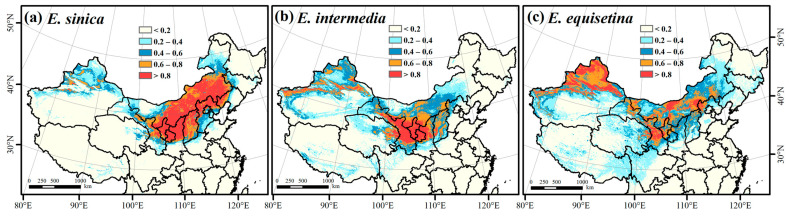
Prediction of the spatial distribution of *E. sinica* (**a**), *E. intermedia* (**b**), and *E. equisetina* (**c**) in China based on the ensemble model.

The highly suitable area (0.6–0.8) and most suitable area (>0.8) were selected as important suitable habitats (ISHs) for the three *Ephedra* herbs. According to the results of the ensemble model in Biomod2 (Figure 2), the ISH areas of *E. sinica* were mainly distributed in central and eastern Inner Mongolia, western Liaoning, northern Hebei, northern Shanxi, northern Shaanxi, Ningxia, and central Gansu, with an area of approximately 133.40 × 10^4^ km^2^ (Figure 2a). Central Inner Mongolia, northern Shanxi, northern Shaanxi, Ningxia, central and eastern Gansu, northern Xinjiang, and eastern Qinghai were the ISH areas for *E. intermedia*, with an area of 89.84 × 10^4^ km^2^ (Figure 2b). The ISH areas of *E. equisetina* were mainly distributed in central and western Inner Mongolia, northern Hebei, northern Shanxi, northwestern Shaanxi, Ningxia, central Gansu, and northern Xinjiang, with an area of approximately 120.41 × 10^4^ km^2^ (Figure 2c). *E. sinica* had the widest range of important suitable habitats, followed by *E. equisetina*, and the range of *E. intermedia* was the smallest.

**Figure 3:**
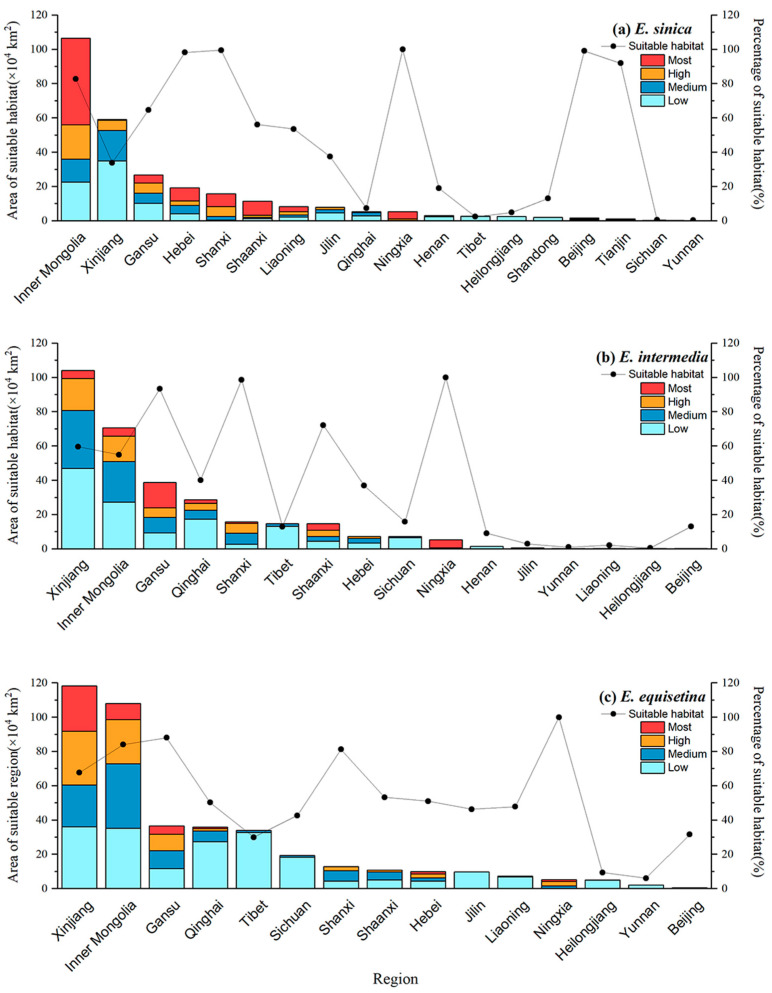
Suitable habitat areas for *E. sinica* (**a**), *E. intermedia* (**b**), and *E. equisetina* (**c**) in China.

We also carried out statistics and analysis on the area of three kinds of *Ephedra* herbs in different administrative regions with different levels of suitability (Figure 3). As shown in Figure 3a, Inner Mongolia had the largest total suitable area for *E. sinica*, with approximately 106.53 × 10^4^ km^2^, followed by Xinjiang and Gansu, with 59.16 × 10^4^ km^2^ and 26.81 × 10^4^ km^2^, respectively. Although the total area of suitable areas in Xinjiang was relatively high, most of these sites had low suitability and were not recommended for the growth of *E. sinica*. According to the percentage of suitable areas (more than 80%) and the area of the ISH, Inner Mongolia, Gansu, Hebei, Shanxi, Shaanxi, and Ningxia were most suitable for the development of *E. sinica*. In Figure 3b, the total suitable area for *E. intermedia* was the highest in Xinjiang, with approximately 104.16 × 10^4^ km^2^, followed by Inner Mongolia and Gansu, with values of 70.70 × 10^4^ km^2^ and 38.72 × 10^4^ km^2^, respectively. The percentage of suitable regions and ISH areas were evaluated, and Xinjiang, Gansu, Inner Mongolia, Shaanxi, and Ningxia were most suitable for planting *E. intermedia*. In Figure 3c, the total suitable area for *E. equisetina* was the highest in Xinjiang (118.25 × 10^4^ km^2^), followed by Inner Mongolia (108.03 × 10^4^ km^2^). The total suitable area was high in Qinghai, Tibet, and Sichuan, but the proportion of areas with low suitability was high, making it unsuitable for the growth and survival of *E. intermedia*. However, Xinjiang, Inner Mongolia, Gansu, and Ningxia had large ISH areas for *E. equisetina*, accounting for a relatively high proportion.

Through a comprehensive ISH evaluation, the proportion of suitable areas and actual local development were used to obtain the final suitable area for the development of three kinds of *Ephedra*. Inner Mongolia had the widest suitable habitat of *E. sinica*, accounting for more than 80%. The eastern part of Inner Mongolia was the most suitable area for the conservation and restoration of *E. sinica*, followed by northern Hebei, northern Shanxi, northern Shaanxi, Ningxia, and central Gansu. The ISH area for *E. intermedia* was the largest in Gansu, and the middle and eastern parts of Gansu were the most suitable areas as priority sites of *E. intermedia*, followed by central Inner Mongolia, Ningxia, and northern Xinjiang. The ISH area for *E. equisetina* was the most extensive in Xinjiang, and the northern part was the most suitable area for the conservation and restoration of this species, followed by the central and western parts of Inner Mongolia and central parts of Gansu.

## 3. Discussion

Using the ensemble model on the Biomod2 platform, this study investigated the potential distribution of three *Ephedra* herbs. Based on occurrence records and environmental factors, ensemble models predicted the habitat suitability maps for *E. sinica*, *E. intermedia*, and *E. equisetina*, with excellent performance measured by the AUC, TSS, and kappa values. As a result, we believe our model results are robust and adequate for constructing the overall suitable habitat distribution of *Ephedra* herbs in China.

### 3.1. Model Results and Verification

According to Flora of China and previous studies, *E. sinica* is mainly distributed in Liaoning, Inner Mongolia, Hebei, Shanxi, Shaanxi, Shandong, Gansu, Qinghai, and Xinjiang; *E. intermedia* is distributed in Liaoning, Inner Mongolia, Hebei, Shandong, Shanxi, Shaanxi, Gansu, Ningxia, Qinghai, Xinjiang; and *E. equisetina* is found in Inner Mongolia, Hebei, Shanxi, Shaanxi, Gansu, Ningxia, Xinjiang [38,39], which was very similar to the ISH range detected in our research. This similarity verifies the accuracy of the results of ensemble models on the Biomod2 platform in predicting species distribution. Furthermore, some researchers used the MaxEnt model to predict the suitable areas for the three *Ephedra* herbs and discovered that *E. sinica* was distributed mainly in central and eastern Inner Mongolia, as well as in eastern Gansu. *E. intermedia* was primarily found in central Gansu Province, with sporadic occurrences in eastern Qinghai and Xinjiang. *E. equisetina* was found in western Inner Mongolia, central Gansu, and northern Xinjiang [40,41], with a much smaller range than that reported in Flora of China and previously recorded. Obviously, the Biomod2 platform is better suited for predicting the suitable distribution habitat of the three species, whereas the MaxEnt model is overly cautious.

Different models have different algorithms and simulation processes, and the prediction results will thus be different [42]. The AUC, TSS, and kappa metrics were chosen as the test values to evaluate the model in our study, and the values were used to effectively evaluate and compare model performance. In single models, RF, GBM, and GLM performed well in predicting the potential distribution of *E. sinica*, *E. intermedia*, and *E. equisetina*, while SRE performed the worst. The accuracy of the ensemble models after screening and integration was higher than that of all single models, which can better predict the range of suitable areas for *Ephedra* resources. This result was similar to that in previous studies on species such as *Salvia miltiorrhiza* Bunge, *Paeonia lactiflora* Pall, and Plateau pika *(Ochotona curzoniae)* [43,44,45]. However, due to the diversity of species growth characteristics and calculation parameters, the matching degree between niche models and species cannot rely only on simple test values. In the future, we should also consider other indicators comprehensively to test the model [46].

### 3.2. Effects of Environmental Variables on Three Ephedra Herbs

Climate, topography, and soil play key roles in plant survival, especially for species in arid areas with harsh habitats. *Ephedra* herbs are xerophytic plants with perennial roots persisting in the soil for many years. Appropriate temperature and water are the basic conditions for the physiological activities of *Ephedra* herbs. Furthermore, topographic factors such as slope, aspect, and elevation, affect plant growth by influencing regional temperature, hydrology, and soil. Previous studies showed that precipitation in the warmest season was a key environmental factor affecting the distribution of *E. foliata*, and the average annual temperature was the main driving force affecting the distribution of *E. gerardiana* [47]. Soil was the main factor influencing the distribution of *E. strobilacea* [48]. In addition, for the co-occurring species of *Ephedra*, *Rosa arabica* Crep., average annual precipitation, elevation, and average annual temperature were important drivers determining its distribution [49]. Similar to previous findings [41], the results of our study revealed that average annual precipitation was the most important environmental factor influencing the distribution of *E. sinica*, elevation was the most important driver influencing the distribution of *E. intermedia*, and *E. equisetina* was most affected by vapor pressure. In summary, topographic and climatic factors are important factors affecting the distribution of *Ephedra*.

Our study found that there were large areas of low-suitability habitats for these three *Ephedra* in several provinces, such as Yunnan and Tibetan autonomous regions, which indicated that *Ephedra* herbs had strong adaptability to the environment. However, because of the complexities of the impact of environmental factors on plants, it was difficult to obtain all of the environmental variables that affect the distribution of these three *Ephedra* herbs and to precisely define their suitability areas. Furthermore, when applying the results to actual planting and restoration, we must consider land occupation as well as the impact of the surrounding environment, such as water quality, vegetation coverage, and human activities. In this study, for example, the suitable areas for *E. sinica* in Beijing and Tianjin were relatively high, but the two cities have more construction land, which is not suitable for the planting and development of *E. sinica*.

### 3.3. Conservation Strategies for Ephedra

Our research identified suitable habitats for three *Ephedra* herbs by constructing ensemble models to predict the potential distribution of *E. sinica*, *E. intermedia* and *E. equisetina*. In the suitable habitat areas of the three species, the protection of *Ephedra* herbs resources should be strengthened. Relevant policies and measures to prohibit the destruction of wild *Ephedra* herbs should be issued, and wild resources can be protected in situ by dividing protected areas [50]. In addition, encouraging the artificial cultivation of *Ephedra* herbs and introducing wild *Ephedra* resources into cultivation are important methods that can be used to protect important endangered resources. *E. sinica* had been artificially introduced for many years, but the other two species were rarely introduced. The government within the scope of important suitable habitat should encourage local farmers to cultivate *E. sinica*, *E. intermedia*, and *E. equisetina*. Meanwhile, farmers’ enthusiasm for planting is affected by market price fluctuations, and one-time planting is easily occurred, resulting in an unstable supply of *Ephedra* medicinal materials [51]. Given the circumstances described above, the government can encourage farmers’ enthusiasm for *Ephedra* cultivation through policy and economic means, promote the balance of planting three *Ephedra* herbs, and promote the protection of wild *Ephedra* resources. Meanwhile, proper harvesting methods are advantageous to the long-term utilization of *Ephedra* resources. *Ephedra* herbs should be harvested in the middle of the branches or near the head, as harvesting close to the root can kill the entire plant [52]. Therefore, farmers must be trained in harvesting to ensure that *Ephedra* can be regrown the following year and that *Ephedra* resources are not depleted.

## 4. Material and Methods

### 4.1. Occurrence Collection

**Figure 4:**
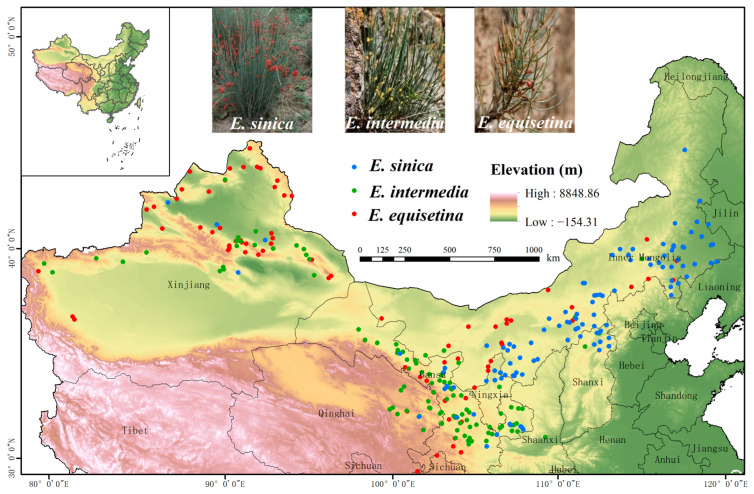
Occurrence of *E. sinica*, *E. intermedia*, and *E. equisetina* in China.

The Global Biodiversity Information Network Database (GBIF, http://www.gbif.org/ accessed on 1 January 2022) China Digital Herbarium (CVH, http://www.cvh.org.cn/ accessed on 1 January 2022) and previous studies [53,54,55] were used to collect distribution data for *E. sinica*, *E. intermedia*, and *E. equisetina* in China for our study. Google Maps was used to locate and obtain coordinate information for samples that lacked geographic coordinate data, and we removed data for which geographic coordinates could not be obtained as well as duplicate data. Using the ArcGIS fishing net tool, we filtered the data in 10 km × 10 km cells to ensure that each *Ephedra* species had only one sample point in each cell. This process could effectively avoid overfitting the model. Finally, a total of 109, 113, and 85 occurrence records of *E. sinica*, *E. intermedia*, and *E. equisetina*, respectively, were obtained for constructing ensemble models (Figure 4). The three *Ephedra* herbs occurrence data were output as longitude and latitude coordinates and saved as CSV files for subsequent analysis. In addition, when using Biomod2 to analyze the potential habitat of species, the species’ nonexistence points must be obtained, and these data are typically difficult to obtain. Therefore, we used the model’s default method ‘random’ to generate pseudo-absence points for the three species, and parallel screening was performed three times [43].

### 4.2. Environmental Parameters

*Ephedra* herbs grow in sandy soil with good air permeability and are mainly found in dry wastelands, riverbeds, and grasslands in arid and semiarid areas. We used three groups of environmental variables based on the distribution characteristics of *Ephedra* herbs: climate factors, soil factors, and topographic factors. Climate factors include solar radiation (srad), vapor pressure (vapr), and 19 bioclimatic variables (bio1~bio19), all of which are standard annual average data from 1970 to 2000 and can be downloaded from the World Climate Database (http://worldclim.org/ accessed on 1 December 2021). Soil factors included soil types (soil), soil particle-size distribution dataset (clay1, clay2, sand1, sand2) [56], and soil quality data (sq1~sq7). The Resource and Environmental Science and Data Center (https://www.resdc.cn/ accessed on 11 June 2022) provided the soil type data. The National Qinghai Tibet Plateau Scientific Data Center (http://data.tpdc.ac.cn/zh-hans/ accessed on 10 March 2022) provided a soil particle-size distribution dataset. The World Soil Database (https://www.fao.org/soils-portal/ accessed on 12 March 2022) provided soil quality data. Elevation (ele), slope (slop), and aspect (asp) were topographic factors, with elevation data derived from the ENVIREM dataset (Environmental Rasters for Ecological Modeling, https://envirem.github.io/ accessed on 12 March 2022)), and we extracted the slope and aspect variables from altitude data by using ArcGIS [57].

These environmental parameters were preprocessed to a general spatial resolution of 30″ latitude/longitude (ca. 1 km^2^ at ground level). We used ArcGIS to extract environmental variables within the study area and output them in ASCII format to construct the model. Since many environmental factors in the same group are calculated from the same set of basic data, the model inevitably has multicollinearity, which leads to a model test value that is too high, causing overly optimistic results [58]. As a result, we used the “corrplot” package in R to analyze the correlation coefficient^®^ between each pair of variables and retained variables that were easy to explain and had a high contribution rate when the |r| between variables was greater than 0.75 [30]. Finally, 20 variables were chosen for the next step for *E. sinica* after removing highly correlated environmental variables; additionally, 17 variables were chosen for *E. intermedia*, and 19 variables were chosen for *E. equisetina* (Table 2).

### 4.3. Model Implementation and Evaluation

We used models in Biomod2 to build ensemble models, and the applicability of different models to the three species was evaluated by calculating the accuracy of the model results, and the optimal ensemble model for each species was constructed. 

The occurrence data and corresponding environmental data for *E. sinica*, *E. intermedia* and *E. equisetina* were input into the Biomod2 platform successively, and the distribution data were divided into two parts: 75% of data were randomly selected for modeling, and the remaining 25% of data were used to test the model results. Three sets of pseudo-nonexistent points were randomly generated, and the model was run 10 times. Therefore, 300 single-model running results (10 × 3 × 10) were generated for each *Ephedra* species.

To evaluate the accuracy and quality of the predictions, area under the curve (AUC), true skill statistics (TSS), and Cohen’s kappa coefficient (kappa) were used in our study. The model is prone to over-dependence when there is only a single evaluation index used to evaluate the model, but different indexes have different responses to diagnostic thresholds and species occurrence distribution rates, and the combination of multiple test values can effectively avoid this situation and better evaluate model performance [59,60,61]. AUC is the area under the receiver operating characteristic (ROC) curve, and it is not affected by the diagnostic threshold and species occurrence and distribution rate, and the range is (0, 1). The AUC was used to verify and evaluate the accuracy and robustness of the model. When the AUC value was above 0.9, the model indicated excellent performance, whereas it was not better than random when below 0.5. TSS has the ability to distinguish between “TRUE” and “FALSE” results, which can effectively avoid the unimodal curve response to the incidence of species. However, it is susceptible to the threshold, and the range is (0, 1); Equation (1) was used to calculate TSS as follows:

(1) $$TSS=\frac{ad-bc}{\left(a+c\right)\left(b+d\right)}$$

where a refers to the number of true positives, b refers to the number of false positives, c refers to the number of false negatives, and d refers to the number of true negatives. Additionally, Kappa was used to predict the accuracy rate relative to random occurrence. It is affected by the incidence rate and threshold, and the range is (0, 1), and its calculation formula is as follows:

(2) $$kappa=\frac{P_{o}-P_{e}}{1-P_{e}},\;\;\;P_{o}=P\cdot Sn+(1-P)\cdot Sp,\;\;\;P_{e}=-2(Sn+Sp-1)P(1-P)+P_{o}$$

where *P*, *Sn*, and *Sp* are the prevalence, sensitivity, and specificity, respectively, *P_o_* is the observed accuracy and *P_e_* is the accuracy expected to occur by chance [62]. The closer the values of AUC, TSS, and kappa are to 1, the better the result was, and the more accurate the prediction of species distribution was. Values further from one indicate a result that is closer to a random estimate.

In our study, AUC, TSS, and kappa were all greater than 0.9, meeting the criteria for screening the results of single models to construct the three combined models. Furthermore, we used a jackknife test to determine the relative importance of the explanatory variables. The potential distribution areas of the three *Ephedra* species are obtained after running the ensemble model, and the results were projected and output to ASCII format for further analysis in ArcGIS. We reclassified the potential distribution area using ArcGIS, converted the grid value to a range of 0–1, and divided the model-predicted distribution area into five grades: unsuitable (0~0.2), low suitability (0.2~0.4), medium suitability (0.4~0.6), high suitability (0.6~0.8), and most suitable (0.8~1) [63,64].

## 5. Conclusions

The ensemble model on the Biomod2 platform was used to simulate and predict the potential suitable habitats of *E. sinica*, *E. intermedia*, and *E. equisetina*, and the effects of environmental factors on the three *Ephedra* herbs were discussed. In the model evaluation, RF, GBM, and GLM demonstrated the best performance among single models. However, the ensemble model improved the prediction accuracy and more accurately simulated the potential suitable habitats of species compared to the single model. We analyzed key environmental variables affecting the distribution of the three *Ephedra* herbs and discovered that average precipitation was the key environmental factor affecting the distribution of *E. sinica*, elevation was the most important environmental factor affecting the distribution of *E. intermedia*, and vapor pressure was the primary factor affecting the distribution of *E. equisetina*. The important factors affecting all three *Ephedra* herbs were annual mean precipitation and elevation. The three *Ephedra* herbs were mostly found in Northwest China. The central and eastern parts of Inner Mongolia were the most suitable development areas for *E. sinica*, the central and eastern parts of Gansu were the most suitable habitats for *E. intermedia*, and northern Xinjiang was the most suitable for the growth of *E. equisetina*. The findings of our study provide some guidance for *Ephedra* herbs cultivation and protection and promote the long-term development of grassland and desert ecosystems.

## Figures and Tables

**Table 1:** The average AUC, TSS, and kappa values of the single models and ensemble models.

Model	*E. sinica*			*E. intermedia*			*E. equisetina*		
	AUC	TSS	Kappa	AUC	TSS	Kappa	AUC	TSS	Kappa
ANN	0.83	0.61	0.61	0.84	0.64	0.64	0.65	0.65	0.84
CTA	0.83	0.64	0.64	0.85	0.70	0.70	0.57	0.57	0.79
FDA	0.87	0.72	0.72	0.89	0.73	0.73	0.67	0.67	0.86
GAM	0.82	0.64	0.64	0.90	0.72	0.72	0.79	0.59	0.59
GBM	0.92	0.77	0.77	0.93	0.78	0.78	0.91	0.76	0.76
GLM	0.87	0.72	0.72	0.91	0.74	0.74	0.83	0.64	0.64
MARS	0.89	0.75	0.75	0.91	0.74	0.74	0.83	0.62	0.62
MaxEnt	0.76	0.50	0.50	0.76	0.51	0.51	0.76	0.50	0.50
RF	0.93	0.78	0.78	0.93	0.78	0.78	0.91	0.76	0.76
SRE	0.72	0.43	0.43	0.74	0.47	0.47	0.66	0.32	0.32
Ensemble	0.97	0.84	0.79	0.98	0.87	0.81	0.98	0.91	0.87

**Table 2:** Environmental variables used or not used in the model.

Variable Type	Code (Unit)	Description	Variables Used in Modeling		
			*E. sinica*	*E. intermedia*	*E. equisetina*
Climatic variables	bio1 (°C)	Annual mean air temperature	√	√	√
	bio2 (°C)	Mean diurnal temperature range (max. temp–min. temp)	√		√
	bio3	Isothermality	√	√	√
	bio4 (°C)	Temperature seasonality		√	
	bio5 (°C)	Max temperature of warmest month			
	bio6 (°C)	Min temperature of coldest month			
	bio7 (°C)	Temperature annual range	√		√
	bio8 (°C)	Mean temperature of wettest quarter	√		
	bio9 (°C)	Mean temperature of driest quarter			
	bio10 (°C)	Mean temperature of warmest quarter			
	bio11 (°C)	Mean temperature of coldest quarter			
	bio12 (mm)	Annual precipitation	√	√	√
	bio13 (mm)	Precipitation of wettest month			
	bio14 (mm)	Precipitation of driest month		√	√
	bio15 (mm)	Coefficient of variation of precipitation	√	√	√
	bio16 (mm)	Precipitation of wettest quarter			
	bio17 (mm)	Precipitation of the driest quarter	√		
	bio18 (mm)	Precipitation of warmest quarter			
	bio19 (mm)	Precipitation of coldest quarter			
	Srad (kJ· m^−2^·d^−1^)	Solar radiation	√	√	√
	Vapr (hPa)	Vapor pressure	√	√	√
Soil variables	soil	Soil type	√	√	√
	clay1	Topsoil Clay Fraction (0–30 cm)	√		√
	clay2	Subsoil Clay Fraction (30–100 cm)		√	√
	sand1	Topsoil Sand Fraction (0–30 cm)	√	√	√
	sand2	Subsoil Sand Fraction (30–100 cm)			
	sq1	Nutrient availability	√	√	√
	sq2	Nutrient retention capacity			
	sq3	Rooting conditions	√	√	√
	sq4	Oxygen availability to roots	√		
	sq5	Excess salts	√	√	√
	sq6	Toxicity			
	sq7	Workability (constraining field management)			
Topographical variables	Ele (m)	Elevation above sea level	√	√	√
	Slop (%)	Slope	√	√	√
	asp (degrees)	Aspect	√	√	√

“√” represents environment variables that are used during model execution.

## Data Availability

Not applicable.
